# Health Status and Health Service Utilization among Vietnamese Farmers in a Mountainous Province

**DOI:** 10.3390/ijerph16234768

**Published:** 2019-11-28

**Authors:** Diep Ngoc Nguyen, Long Hoang Nguyen, Cuong Tat Nguyen, Hai Quang Pham, Jongnam Hwang, Giang Thu Vu, Bach Xuan Tran, Carl A. Latkin, Cyrus S. H. Ho, Roger C. M. Ho

**Affiliations:** 1 Institute for Global Health Innovations, Duy Tan University, Da Nang 550000, Vietnam; diep.ighi@gmail.com (D.N.N.); qhai.ighi@gmail.com (H.Q.P.); 2 Center of Excellence in Behavioral Medicine, Nguyen Tat Thanh University, Ho Chi Minh City 700000, Vietnam; longnh.ph@gmail.com (L.H.N.); pcmrhcm@nus.edu.sg (R.C.M.H.); 3 Division of Social Welfare and Health Administration, Wonkwang University, Iksan 54538, Korea; jonhwang416@gmail.com; 4 Center of Excellence in Evidence-based Medicine, Nguyen Tat Thanh University, Ho Chi Minh City 700000, Vietnam; giang.coentt@gmail.com; 5 Institute for Preventive Medicine and Public Health, Hanoi Medical University, Hanoi 100000, Vietnam; bach.ipmph@gmail.com; 6 Bloomberg School of Public Health, Johns Hopkins University, Baltimore, MD 21205, USA; carl.latkin@jhu.edu; 7 Department of Psychological Medicine, National University Hospital, Singapore 119074, Singapore; cyrushosh@gmail.com; 8 Department of Psychological Medicine, Yong Loo Lin School of Medicine, National University of Singapore, Singapore 119228, Singapore; 9 Institute for Health Innovation and Technology (iHealthtech), National University of Singapore, Singapore 119077, Singapore

**Keywords:** Vietnam, farmer, mountainous, health status, quality of life, utilization, self-rated health

## Abstract

Problems of poor health status and low health service use among farmers in mountainous areas have not been fully investigated. A cross-sectional study was conducted in Son La, a mountainous province in Vietnam, to assess the self-rated health and health care service utilization among farmers. Visual analogue scale (VAS) was used to measure the self-rated health. Multivariate Tobit, Poisson, and logistic regression were employed to identify related factors. Among 197 farmers, the mean VAS score was 67.8 (SD = 15.5). Approximately 40% of participants reported health problems, and the most popular morbidity was hypertension—56.4%. There were 28.9% and 50.3% of farmers using inpatient and outpatient treatments in the last 12 months, respectively. Age, educational level, family income, marital status, alcohol use, and source of information have been identified as associated factors with self-rated health status and morbidities, while age, gender, education, and morbidities were related to health service utilization. Data indicated a high proportion of health issues and a high rate of health care service use among farmers in a mountainous area of Vietnam. Adaptable health policies and prevention programs or preventive health services should be implemented regularly in mountainous regions to protect farmers from the onset of morbidities and to enhance their health.

## 1. Introduction

Self-rated health (SRH) status and health service utilization are extensively used as primary indicators to evaluate the efficacy of the public health system in places where there is insufficient health data, especially in low- and middle-income countries [1]. Measuring these indicators can be helpful to assess the health differentiation among diverse population groups. In the previous literature, inequality in SRH and health service utilization have been identified across gender, age, educational level and socioeconomic groups [2,3]. Moreover, some factors have been confirmed that influence health service utilization, such as physical accessibility and quality of services, cultural beliefs and perceptions, gender and age, insurance, and socioeconomic status, together with preferences of patients [4,5,6].

In developing countries, farmers are at a high risk of poor health conditions due to farming risks (physical hard work, pesticides and other toxic chemicals), climate change, or low-quality living conditions [7,8,9,10]. Previous studies in both developed and developing countries revealed that farmers were more likely to suffer from illness (skin, respiratory, neurologic, and kidney problems) and poorer mental health (anxiety/depression, memory loss, dizziness, etc. [11,12,13,14,15,16]). These health consequences could result in low work productivity and increase the economic burden of households [9,17,18]. Farmers living in mountainous areas are particularly vulnerable to low health status due to limited health service accessibility in comparison with non-farmers in similar areas, as well as in urban and rural settings [14,18,19,20,21,22,23,24,25,26]. This problem is attributable to low technology infrastructure [27,28,29], geographical barriers, low educational level, low quality of health care providers, and financial difficulties [4,30,31]. However, since health status and health care utilization have been found to be different among countries, it is necessary for researchers and policymakers to obtain more contextualized evidence about health status and health service utilization among mountainous/remote farmers in different settings. These data could serve as a foundation for developing interventions to enhance farmers’ health outcomes and work productivity.

In Vietnam, health care services are provided by both public and private sectors in all regions [32]. Domestic resources, including a social health insurance scheme and out-of-pocket payment, are the main funding sources for health care [33]. In a previous study, the proportion of mountainous people with health insurance was much higher than those living in rural and urban areas [34]. Similar to other developing countries, Vietnamese people living in mountainous areas suffer from typical problems such as an undeveloped economy, low access to health information and education, and limited public transportation [22,23,24,25,26,35], which, in turn, would adversely impact health status as well as leading to disadvantages in health care access and utilization. Although previous studies have been carried out on health status and health-service utilization in general [2,3] or reproductive health service use among mountainous women [36,37], no single study exists with farmers. Therefore, this study aimed to provide a deeper understanding of the health status and the use of health care services among farmers living in a mountainous province of Vietnam, and to identify associated factors.

## 2. Materials and Methods

### 2.1. Study Design and Participant Recruitment

A cross-sectional study was conducted from August to September 2018 in the Moc Chau district of Son La, a mountainous province in the Northwest of Vietnam. This rural district has a population of 104,730 and covers an area of approximately 1081 km^2^ [38]. Moc Chau is located on the limestone karst system in the Moc Chau plateau with a relatively flat terrain, fertile soil, and cool climate. By 2014, there were 5/15 communes that met the national criteria for health care with 3.5 doctors/10,000 people, 13 patients/10,000 people and one general hospital having 150 inpatient beds [38].

In this study, we applied a formula to estimate a population proportion with specific relative precision. With a confidence level of 95%, expected prevalence of morbidities among farmers residing in mountainous areas was 0.53 [39] and relative precision was 0.13. The required sample size was 194 farmers. First, we prepared a list of total households in the Moc Chau district. We randomly selected 200 households by using computer software. In each household, we randomly chose one family member. The participants who complied with the required criteria were selected to be involved in the study: (1) being 18 years old or above; (2) being at Moc Chau in the time of interviewing; (3) being willing to be involved in the study; (4) being capable of providing information to the interviewers. We excluded participants who suffered from serious illnesses during the recruitment process. A total of 197 respondents consented to be study participants.

### 2.2. Measure and Instruments

A 10 min face-to-face survey was conducted by well-trained researchers. In order to protect participants’ confidentiality, we invited participants to a small private counseling room in the commune health stations where they lived. We also informed respondents about the benefits and drawbacks of volunteering for the study and their decision would not affect their ability to use health-examination services. If they agreed to enroll in the study, respondents provided signed written informed consent.

In order to accord with preferences and culture of participants, a pilot survey of 20 participants of both genders in different age groups was first tested, and only minor changes were made based on this piloting. During the recruitment and the conduct of the study, health staff in commune health stations did not attend. We developed a structured questionnaire with the following information.

#### 2.2.1. Socioeconomic Characteristics

Participants provided information on gender, age, ethnicity, educational level, marital status, monthly family income, and number of household members.

#### 2.2.2. Health Status

Participants’ health status of blood pressure, height, and weight was assessed. Standard blood pressure indices were used to determine high blood pressure, and the cut off for hypertension stage 1 was 130 for systolic and 80 for diastolic; for hypertension stage 2 it was 140 for systolic and 90 for diastolic [40]. Body mass index (BMI) of participants was calculated based on their height and weight measurements. We also asked participants whether they were diagnosed with any chronic diseases in the last 3 months by health professionals. In order to assess self-rated health status among participants, we used the visual analogue scale (VAS) with endpoint varies from 0 (“the worst health you can imagine”) to 100 (“the best health you can imagine”) points in its 20 cm vertical scale [41].

#### 2.2.3. Health Care Utilization and Health Information

The information regarding health care utilization was determined in our study. Participants were asked to report the number of times they attended inpatient and outpatient health visits in the past 12 months, as well as their primary source of health-related information.

### 2.3. Statistical Analysis

Data were analyzed by STATA version 12 (Stata Corp. LP, College Station, TX, USA). Multivariate Tobit and Poisson regressions were employed to identify factors associated with self-rated health and number of morbidities, respectively. Moreover, to determine factors related to health care service use, we used multivariate logistic regression for variables “whether using inpatient/outpatient services in the last 12 months” (Yes/No) and “whether using outpatient services in the last 12 months” (Yes/No); and Poisson regression for variables “number of inpatient treatments in the last 12 months” and “number of outpatient treatments in the last 12 months”. In order to discard non-significant factors, a forward stepwise selection strategy was employed. The log-likelihood ratio test’s *p*-value was set at less than 0.2, which was adjusted as the threshold to select a variable. A *p*-value < 0.05 was considered as statistically significant.

## 3. Results

According to Table 1, the majority of the respondents were an ethnic minority, accounting for nearly 70%. Almost all the respondents were married, while the average age was 44.9 years (SD = 11.8). The average number of family members was 4.5 (SD = 1.8).

Table 2 indicates the health status of Vietnamese farmers in the mountainous province. The proportion of the respondents who had normal weight and blood pressure indicators was 63.5% and 34.4%, respectively. In terms of current health status, approximately 40% of the farmers reported that they had at least one disease. The mean VAS score of the respondents was 67.8 (SD = 15.5).

**Figure 1:**
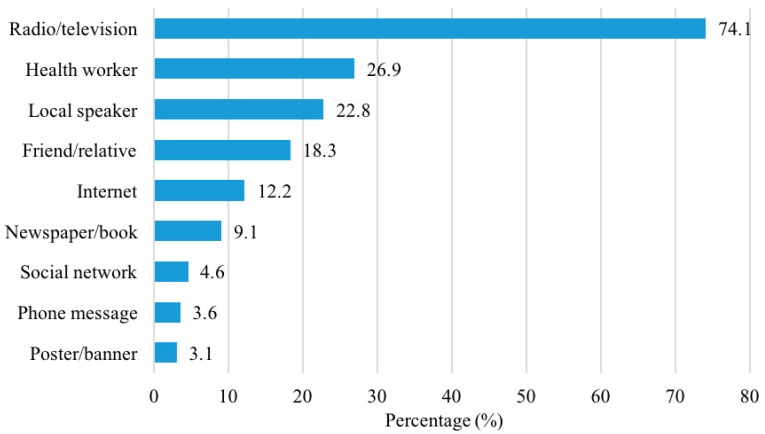
Sources of health information among participants.

Figure 1 illustrates the sources of health information among participants and health information demand. The prevalence of participants who responded that they received health information from radio/television and a health worker source were highest, at 74.1% and 26.9%, respectively. The least common channel was poster or banner, which only accounted for 3.1%.

Table 3 presents the associated factors with health status. It can be seen that among the alcohol-use group, people who had a higher frequency of alcohol use were likely to have lower VAS. However, the using-alcohol groups had relatively higher VAS scores (5.51 to 10.32) compared to the non-using alcohol group. Individuals who were single had relatively lower VAS scores compared to those who had a spouse or partner.

Table 4 shows the associated factors with health care service utilization. Age and education were significantly associated with number of inpatient and number of outpatient treatments. Status of health such as weight status and comorbidities were positively associated with the number of treatments received.

## 4. Discussion

Our study contributes to the existing literature on health status and health care service use among vulnerable populations by exploring the situation of farmers in mountainous settings of Vietnam. Findings from this study showed, on average, significantly poor SRH, a high number of health problems, and a high rate of health care service utilization among farmers in a mountainous setting of Vietnam. Several socioeconomic characteristics have been pointed out as associated factors with these outcomes, suggesting important implications to address the health-inequality gap in this population. 

Our sample had a relatively high rate of health problems, particularly arthritis (20.3%), transient ischemic attack (TIA) (16.8%), and hypertension (56.4%). These rates were much higher compared to other populations residing in different settings of Vietnam such as urban people (arthritis rate was 14.5%) [42], and the general population (TIA rate of 0.01% [43,44], and hypertension rate of 25.1%) [45]. There are several possible explanations for these differences. First, pesticides and farming chemicals could bring high risk of illness during agricultural activity. Second, the age of our participants was higher than those in previous studies. Morbidity rates in our farmers were also greater than those in other countries such as Canada [46], Nigeria [47] or Portugal [43], suggesting a high need for medical care in farmer populations in Vietnamese mountainous settings. Moreover, we found that those reporting friends/relatives and health workers as primary health information sources reported the lowest number of health problems. A previous study showed that about 54% of households in Vietnam considered health information provided by health workers as highly valued for their health issues [48]. This finding underlined a potentially critical role of health workers in delivering health messages to improve the health outcomes of mountainous farmers in Vietnam.

Our participants also had lower self-rated health status (VAS score 67.8) compared to that of the general population (VAS score 77–90) [49]. VAS scores in our sample were also lower than that of farmers in China (mean score 83.59) [50], and in the United Kingdom (mean score 79) [51]. A high frequency of alcohol use was found to be associated with high QOL. A study in Vietnam showed evidence that alcohol-dependent subjects had a higher quality of life score, and “controlled” (or minimal) drinking could improve quality of life [52]. It also is possible that those with health problems have ceased drinking. Moreover, being single was related to lower SRH, which might due to the fact that people who lived with others felt safer and were less depressed. Prior research in Vietnamese rural regions indicates that individuals living with their families had higher QOL in comparison with those living alone [53,54]. Our studies were also in line with the literature showing that higher age and lower income are associated with lower SRH [55,56,57].

In this study, we found high rates of health care service utilization, representing a high need for health care among farmers in a mountainous region. Our rates were significantly higher than those among the general Vietnamese population (8.1% used inpatient treatments and 37.1% used outpatient treatments in the last 12 months) [58], as well as other occupational populations such as industrial workers, with only 38.8% of migrant workers using health care services [59]. This discrepancy could be attributed to health literacy among different settings. Indeed, people in mountainous regions have lower educational levels, resulting in insufficient health-related knowledge for protecting themselves; thus, their needs regarding health care service utilization might be higher than other populations. Moreover, this finding implies good health service access in mountainous farmers, suggesting the need for preventive health care services to reduce the incidence of poor health. Of note, people with secondary education or less had a lower likelihood of inpatient service use but higher outpatient use than those with lower levels of education. This difference may be due to a greater awareness of their morbidities, and hence they would use health care services when necessary. Alternatively, they may seek services before there is a need for inpatient treatments. However, we also found that the higher the education, the less frequency of outpatient use, which might be attributed to self-treatment or less morbidity. A previous study highlighted findings that 40–60% people in Vietnam depended on self-treatment when family members had health problems, and the higher the level of education they obtained, the more frequently self-medication occurred [60].

Several implications can be drawn from this study. First, it is necessary to implement more frequent mobile health screening programs to collect data on the health status of farmers in difficult-to-reach regions in order to fill the gap in health status and utilization among different groups in Vietnam [5,37,61,62]. Second, since the rate of health care service use in our population was higher than that in the general population, but the self-rated health and morbidity prevalence were also higher, more preventive measures such as educational programs should be considered to enhance their health to prevent the occurrence of diseases. These recommendations could partly enhance the current situation and lessen the predicament that farmers in mountainous regions are facing.

The study has several limitations. First, our study had the common limitation of a cross-sectional study design, which impedes the ability to determine the causal relationship between independent and dependent variables. Secondly, participants of our study might not be representative of all farmers in mountainous areas of Vietnam. Therefore, it is necessary to implement further research in other regions to achieve suitable solutions in decreasing the gap in health status and health care service use among different settings. Third, lacking control groups such as non-farmers living in similar settings or healthier people migrating to the cities might constrain our conclusion about the vulnerability of farmers in mountainous areas. Finally, information about morbidities was self-reported, which might lead to recall bias. Moreover, the prevalence of diseases might be underestimated because some participants might have certain conditions but not have been diagnosed.

## 5. Conclusions

In conclusion, data indicated a high proportion of health issues and a high rate of health care service use among farmers in a Vietnamese mountainous area. Adaptable health policies and prevention programs or preventive health services should be implemented regularly in mountainous regions to protect farmers from the onset of morbidities and enhance their health.

## Figures and Tables

**Table 1:** Socio-economic status of respondents (*n* = 197).

Characteristics	*n*	%
Age group		
<30	19	9.6
30–40	54	27.4
41–50	61	31.0
>50	63	32.0
Ethnicity		
Kinh	61	31.0
Other	136	69.0
Education		
Under secondary	82	42.1
Secondary	77	39.5
Above secondary	36	18.5
Marital status		
Having a spouse/partner	178	90.8
Single	18	9.2
Alcohol use		
Never	93	47.7
Monthly or less	33	16.9
2–4 times a month	17	8.7
2–3 times a week	33	16.9
4 or more times a week	19	9.7
	**Mean**	**SD**
Age	44.9	11.8
Number of family members	4.5	1.8
Monthly household income (USD)	253.7	211.7

**Table 2:** Health status of Vietnamese farmers in a mountainous province.

Characteristics	*n*	%
Body mass index categories		
Underweight	8	5.1
Normal	99	63.5
Overweight	49	31.4
Blood pressure categories		
Normal	67	34.4
Elevated	18	9.2
Hypertension stage 1	52	26.7
Hypertension stage 2	58	29.7
Morbidity		
Stroke	4	2.0
Transient ischemic attack	33	16.8
Diabetes	11	5.6
Parkinson’s disease	1	0.5
Arthritis	40	20.3
Blood lipid disorders	26	13.2
Cancer	7	3.6
Number of morbidities		
Do not have a disease	118	59.9
Have one disease	50	25.4
Have more than one disease	29	14.7
Using inpatient services	57	28.9
Using outpatient treatments	99	50.3
	**Mean**	**SD**
VAS	67.8	15.5
Number of inpatient treatments	0.5	1.2
Number of outpatient treatments	1.7	3.1

**Table 3:** Associated factors with self-rated health status.

Characteristics	VAS Score		Number of Morbidities	
	Coef	95% CI	Coef	95% CI
Age group (vs. <30)				
30–40			0.65 *	−0.01; 1.31
41–50	−8.05 ***	−12.97; −3.13	0.93 ***	0.36; 1.51
Ethnicity (Others vs. Kinh)			−0.30	−0.74; 0.14
Marital status (Having spouse/partner vs. Single)	7.48 **	0.27; 14.70	−0.72 *	−1.49; 0.04
Body mass index categories (Overweight/obesity vs. Normal)	3.48	−1.50; 8.46		
Alcohol use (vs. Never)				
Monthly or less	10.32 ***	3.98; 16.65		
2–3 times a week	8.56 ***	2.59; 14.54		
Four or more times per week	5.51	−2.38; 13.40		
Income quintiles (vs. Poorest)				
Poor	1.00	−6.26; 8.27	0.58	−0.21; 1.37
Middle	7.30 **	0.84; 13.76	−0.09	−0.88; 0.71
Rich	6.92 **	0.41; 13.44	0.73 **	0.09; 1.36
Richest	6.89	−1.35; 15.14	0.12	−0.91; 1.15
Source of health information				
Friend/relative (Yes vs. No)			0.61 **	0.05; 1.17
Poster/Banner (Yes vs. No)			1.18 ***	0.44; 1.91
Phone message (Yes vs. No)	7.45	−6.09; 20.98	1.20 ***	0.60; 1.80
Newspaper/book (Yes vs. No)	−5.81	−13.07; 1.46		
Health worker (Yes vs. No)			0.64 **	0.10; 1.19
Social network site (Yes vs. No)	−9.66 *	−19.68; 0.36		
Other (Yes vs. No)			0.95 *	−0.03; 1.94

*** *p* < 0.01, ** *p* < 0.05, * *p* < 0.1.

**Table 4:** Associated factors with health care service utilization.

Characteristics	Using Inpatient Treatments		Number of Inpatient Treatments		Using Outpatient Treatments		Number of Outpatient Treatments	
	OR	95% CI	Coef	95% CI	OR	95% CI	Coef	95% CI
Age group (vs. <30)								
30–40	2.54 *	0.97; 6.68			3.67 *	0.87; 15.54	1.26 ***	0.34; 2.18
41–50	2.05	0.80; 5.25			4.78 **	1.12; 20.35	1.32 ***	0.40; 2.24
>50					4.39 **	1.03; 18.65	1.59 ***	0.69; 2.50
Gender (Female vs. Male)			0.10	−0.37; 0.57	2.19 **	1.12; 4.30	0.21 *	−0.04; 0.47
Education (vs. Under secondary)								
Secondary	0.40 **	0.18; 0.91					0.44 ***	0.16; 0.71
Above secondary							−0.50 **	−0.92; −0.07
Body mass index categories (Overweight/obesity vs. Normal)	2.43 **	1.13; 5.20	0.42 *	−0.07; 0.91			0.27 **	0.01; 0.53
Blood pressure categories (vs. Normal)								
Elevated			−0.36	−1.15; 0.43				
Hypertension stage 1			−0.92 **	−1.63; -0.21				
Hypertension stage 2			−0.41	−0.99; 0.17				
Number of morbidities (vs. No disease)								
One disease			0.74 **	0.13; 1.35			0.88 ***	0.58; 1.18
More than one disease	2.85 **	1.05; 7.76	1.59 ***	1.05; 2.13	2.50 *	0.91; 6.89	0.99 ***	0.66; 1.31

*** *p* < 0.01, ** *p* < 0.05, * *p* < 0.1.
